# Genome-wide identification of NF-Y gene family in maize (*Zea mays L.*) and the positive role of ZmNF-YC12 in drought resistance and recovery ability

**DOI:** 10.3389/fpls.2023.1159955

**Published:** 2023-05-17

**Authors:** Liru Cao, Chenchen Ma, Feiyu Ye, Yunyun Pang, Guorui Wang, Abbas Muhammad Fahim, Xiaomin Lu

**Affiliations:** ^1^ Grain Crops Research Institute, Henan Academy of Agricultural Sciences, Zhengzhou, China; ^2^ The Shennong Laboratory, Zhengzhou Henan, China; ^3^ Department of Plant Breeding and Genetics, Faculty of Agricultural Sciences and Technology, Bahauddin Zakariya University, Multan, Pakistan

**Keywords:** nuclear factor Y, maize, bioinformatics, transcriptional activator, drought resistance, recovery ability

## Abstract

Nuclear factor Y (NF-Y) genes play important roles in many biological processes, such as leaf growth, nitrogen nutrition, and drought resistance. However, the biological functions of these transcription factor family members have not been systematically analyzed in maize. In the present study, a total of 52 *ZmNF-Y* genes were identified and classified into three groups in the maize genome. An analysis of the evolutionary relationship, gene structure, and conserved motifs of these genes supports the evolutionary conservation of NF-Y family genes in maize. The tissue expression profiles based on RNA-seq data showed that all genes apart from *ZmNF-Y16*, *ZmNF-YC15*, and *ZmNF-YC17* were expressed in different maize tissues. A weighted correlation network analysis was conducted and a gene co expression network method was used to analyze the transcriptome sequencing results; six core genes responding to drought and rewatering were identified. A real time fluorescence quantitative analysis showed that these six genes responded to high temperature, drought, high salt, and abscisic acid (ABA) treatments, and subsequent restoration to normal levels. *ZmNF-YC12* was highly induced by drought and rewatering treatments. The ZmNF-YC12 protein was localized in the nucleus, and the Gal4-LexA/UAS system and a transactivation analysis demonstrated that *ZmNF-YC12* in maize (*Zea mays L.*) is a transcriptional activator that regulates drought resistance and recovery ability. Silencing *ZmNF-YC12* reduced net photosynthesis, chlorophyll content, antioxidant (superoxide dismutase, catalase, peroxidase and ascorbate peroxidase) system activation, and soluble protein and proline contents; it increased the malondialdehyde content, the relative water content, and the water loss rate, which weakened drought resistance and the recoverability of maize. These results provide insights into understanding the evolution of ZmNF-Y family genes in maize and their potential roles in genetic improvement. Our work provides a foundation for subsequent functional studies of the NF-Y gene family and provides deep insights into the role of the *ZmNF-YC12* regulatory network in controlling drought resistance and the recoverability of maize.

## 1 Introduction

Maize is a major food and feed crop that is susceptible to various environmental stresses, the most important of which is drought, as it influences the growth and development of crops (Nakashima and Yamaguchi-Shinozaki, 2013). Climate change and the increasing world population pose serious challenges that necessitate crop improvement. The breeding of drought-tolerant maize and improving the crop yield using biotechnological methods are of great strategic importance.

The nuclear factor Y (NF-Y), known as HAP (heme activator protein) or CBF (CCAAT binding factor), is a complex composed of three different subunits, NF-YA, NF-YB and NF-YC. The NF-Y complex has been studied broadly in the animal system, and the highly conserved domain has been shown to play an important role in the interaction between the NF-Y transcription factor protein and DNA (Deoxyribonucleic Acid) (Mantovani, 1999). Unlike in yeast and mammals, where each subunit is encoded by one gene, plants have numerous NF-Y subunit genes (Laloum et al., 2013); 10 genes encode NF-YA, 11 genes encode NF-YB and 7 genes encode NF-YC in rice (Thirumurugan et al., 2008), 10, 11 and 14 in wheat (Stephenson et al., 2007); and 14 18 and 18 in corn (Zhang et al., 2016), respectively. While the NF-YA members of plants are different in structure and length, they all contain a conserved sequence composed of 53 amino acids (Petroni et al., 2012). The NF-Y family genes are known to play important role in regulating plant embryogenesis (Mu et al., 2013) to and response to abiotic stresses. (Ma et al., 2015).

Overexpression of the *AtNF-YB2* gene accelerates the differentiation of Arabidopsis root tip cells and promotes the elongation of primary roots (Ballif et al., 2011). The *OsNF-YB/2/3/4* complex regulates the expression of chlorophyll a/b binding protein-related genes (Miyoshi et al., 2003). The complex formed by *AtNF-YA5*, *AtNF-YB9* and *AtNF-YC9* is jointly involved in the regulation of the expression of chlorophyll a/b binding protein-coding genes in the yellowing seedlings of *Arabidopsis* (Warpeha et al., 2007). Overexpression of the wheat *TaNF-YAl0* gene in Arabidopsis reduces the sensitivity of plants to ABA and increases the plant sensitivity to salt stress (Ma et al., 2015). Overexpression of *AtNF-YAl* in Arabidopsis can improve the sensitivity of plants to salt stress and ABA (Li et al., 2013). Overexpression of *ZmNF-YB2* and *AtNF-YBl* in maize and Arabidopsis can improve the drought resistance of plants (Nelson et al., 2007). In maize, *ZmNF-YA3* enhances drought and high-temperature tolerance by binding to the promoters of *bHLH92*, *FAMA* and *MYC4*, thereby regulating their gene expression (Su et al., 2018). *ZmNF-YA1* and *ZmNF-YB16* regulate the growth, development, and drought resistance of maize (Yang et al., 2022), and *ZmNF-YB16* improves drought resistance and yield by enhancing the photosynthetic and antioxidant capacity of maize (Wang et al., 2018). Overexpression of the *CdtNF-YC1* gene can enhance the drought-resistance and salt-tolerance ability of transgenic Seashore Paspalum plants by increasing the relative water and chlorophyll content of plants (Wu et al., 2018). Compared with wild-type plants, *AsNF-YC8*-verexpressing transgenic tobacco plants showed higher seed germination rates, longer root length and better plant growth under salt and drought stresses (Sun et al., 2017).

At present, research on the function of NF-Y is mostly concentrated in Arabidopsis, rice, and other model plants. Although maize has relatively higher members of NF-Y subunits, no comprehensive study of the NF-Y gene family has been conducted to date, and the underlying mechanisms of ZmNF-Y genes remain largely unknown. In the present study, 52 putative ZmNF-Y genes were identified in maize and subjected to phylogenetic, gene structure, motif, and chromosomal location analyses. In addition, analyses of the tissue-specific and differential expression profiles of maize ZmNF-Y genes under drought and rewatering treatments were conducted. Through weighted gene co-expression network analysis (WGCNA) under various abiotic stresses, six core genes responding to drought and rewatering treatments were screened. Moreover, the transcriptional activity of the *ZmNF-YC12* gene was analyzed by subcellular localization, yeast trans activation system, and Gal4 LexA/UAS system. To verify the drought resistance and growth recoverability of *ZmNF-YC12*, we further silenced its expression through VIGS (virus induced gene silencing) technology to obtain homozygous mutants of *ZmNF-YC12* to identify the function of *ZmNF-YC12* in drought resistance and growth recoverability. Our study provides a platform for the further investigation of the functions of *ZmNF-Y* genes in maize drought stress and rewatering responses, whilst also providing new ideas for drought-resistant molecular breeding.

## 2 Materials and methods

### 2.1 Plant growth conditions and treatment

Maize “B104” (Zea mays L., inbred line) was germinated in a greenhouse for five days with growth conditions of 28°C under a 14-h light/10-h dark cycle. Maize “B104” was subjected to drought stress at the three leaves stage, at soil moisture level 45-48% drought stress were maintained for 5 days. Then the maize were rewatered for three days at soil moisture content above 95%. Leaves and roots of before stress, drought stress and rewatering were collected (denoted as T0Y, T5dY, TR3dY and T0G, T5dG, TR3dG, respectively) and subjected to RNA (Ribonucleic Acid) sequencing (RNA-Seq). The second experiment, we transferred seedings of three-leaf stage to nutrient solution containing 20% PEG6000 (Polyethylene glycol 6000), 42°C, ABA (5 µmol/L) and NaCl (200 mmol/L) conditions. The leaves of maize were harvested at 0, 4, 8, 12, 24, 32, 36, 48, 52, 60, 72, 84, R12, R36 h (“h” stands for the hour, “R” stands for the rewatering.) and stored at −80°C. There were three biological replicates per treatment. Maize inbred line Va35 was grown in green house at 20°C under a 16-h light/8-h dark cycle. Tobacco was grown in green house at 22°C under a 10-h light/14-h dark cycle.

### 2.2 Isolation of RNA and quantitative real-time PCR analysis

A total of 15 genes screened were quantitatively analyzed by quantitative real-time PCR (qRT-PCR) and the primer sequences were listed in **Supplementary Table S1**. We extracted RNA from the leaves of three independent biological replicates for each at 0, 4, 8, 12, 24, 32, 36, 48, 52, 60, 72, 84, R12 and R36 h. The first-strand cDNA (Complementary DNA) was synthesized by a Hifair^®^ II 1st Strand cDNA Synthesis SuperMix (YEASEN, Shanghai, China). Gene-specific primers for qPCR were designed by using Primer5 according to the corresponding sequence and are shown in **Table S4**. Actin 18s was used as an internal control. According to the manufacturer’s instructions, the qPCR analyses were performed using Hieff^®^ qPCR SYBR^®^ Green Master Mix (YEASEN, Shanghai, China) on a Light Cycler 480 instrument (Roche, Basel, Switzerland). Each gene was analyzed for three technical replicates. The relative expression level (2^−ΔΔCt0 h^) of untreated control plants was normalized to 1.

### 2.3 Collection and classification of NY-F transcription factors

The NF-Y domains were screened using Pfam, SMART program and plant transcription factor database (http://planttfdb.gao-lab.org/index.php sp=Zma), and 16, 19, and 17 members of the NF-YA, NF-YB, and NF-YC families were identified, respectively. Using the NCBI Conserved Domain Database (CDD) (https://www.ncbi.nlm.nih.gov/Structure/cdd/wrpsb.cgi) to determine all the retrieved sequences. The molecular weight (MW) and isoelectric point (PI) of the sample were predicted by ProtParam (http://web.expasy.org/protparam/).The amino acid sequences, chromosome positions of these genes and the homologous Sorghum (*Sorghum bicolor (L.) Moench*), Oryza sativa Japonica (*Oryza sativa* L.*subsp*. *Japonica*)) and Arabidopsis were downloaded using Phytozome (https://phytozome-next.jgi.doe.gov/).

### 2.4 Compound phylogenetic tree, additional conserved motifs analysis, cis-elements in the promoter regions and collinearity analysis

Full-length protein sequence alignments of ZmNY-Fs and the homologous sorghum and Arabidopsis family were generated using the MEGA (https://www.megasoftware.net) and then manually adjusted to the alignment. The phylogenetic tree was constructed in MEGA6 by neighbor-joining (NJ) method. We used the online software Multiple Expectation maximization for Motif Elicitation (MEME) program (http://meme-suite.org/tools/meme) to query the motifs of maize NY-F proteins (**Supplementary Table S2**). To determine the evolutionary relationship of NY-F proteins among other species, we used the MCscan algorithm (Tang et al., 2008) to perform collinearity analysis among the NY-F genomes of Maize and Arabidopsis, Sorghum, and Rice. To predict cis-acting regulatory DNA elements (cis-elements) in promoter regions of maize NY-F genes, we used the online software the PLACE website (Available online http://www.dna.affrc.go.jp/PLACE/signalscan.html) to identify putative cis-elements in the 2000 bp genomic DNA sequences upstream of the start codon (ATG).

### 2.5 Microarray-based expression analysis of ZmNY-F genes

We obtained transcriptome data from the maize genome mapping article developed by RNA sequencing and comparative evaluation of transcriptomes based on RNA sequencing and microarrays. In at least one of the 10 tissues, the average gene expression value must be greater than 0 Fragments Per Kilobase Exon model per Million mapped fragments (FPKM).

### 2.6 Transcriptome sequencing using Illumina HiSeqTM 2500 platform

To analyze **e**xpression pattern of *ZmNY-F* genes during drought-rewatering process, total RNA was extracted using TRIzol reagent (Invitrogen, Carlsbad, CA, USA). RNA concentration was measured using a Qubit Fluorometer (Invitrogen) and RNA quality was verified using Agilent 2100 Bioanalyzer (Agilent, Palo Alto, California, USA). The minimum RNA integration value was 8. After total RNA was extracted by TRIzol, RNA molecules with a size of 18-30 nt can be enriched using polyacrylamide gel electrophoresis (PAGE). The Illumina HiSeqTM 2500 platform was used to reverse transcription of the enriched mRNA, amplify and sequence by polymerase chain reaction. High-quality clean reads are obtained by removing those containing adaptors and containing more than 50% of low-quality (Q value ≤ 20) and more than 10% of unknown nucleotides (N) bases. Finally, through mapping reads to the rRNA database using the short reads comparison tool Bowtie2, ribosome RNA (rRNA) mapped reads were removed. The remaining reads are used for transcriptome assembly, and the raw data is compared with the maize inbred line B73V4 database and gene annotation. The pathways of these genes were analyzed using Blast2GO. We used the R package (http://www.r-project.org/) to identify differentially expressed genes (DEGs) samples or groups. We found genes with |log2 (fold change)| ≥ 2 and the false discovery rate (FDR) < 0.05 in a comparison as significant differentially expressed genes. Differential gene expression was enriched by R language heat map software. Correlation coefficients between genes were calculated according to gene expression. To screen key node genes, a correlation coefficient greater than 0.75 has been introduced into version 3.71 Cytoscape software [(https://cytoscape.org)] to construct the gene co-expression network.

### 2.7 Weighted gene coexpression network construction

Using TBtools with the default parameters, we analyzed and constructed a weighted gene co-expression network (WGCNA) of alfalfa under drought stress and rewatering (Chen et al., 2020; Zhao et al., 2021). First, using transcriptome data (drought stress and rewatering) harvested T0Y, T5dY, TR3dY and T0G, T5dG, TR3dG, all expressed genes were screened according to FPKM values (more than 1) for the subsequent analysis. Then, the correlation between different modules and drought stress and rewatering treatment times were obtained by cluster analysis and correlation analysis. Finally, co-expression network drawing was mapped using Cytoscape software (Shannon et al., 2003).

### 2.8 Determination of subcellular localization of ZmNF-YC12

A complete open reading frame (ORF) of *ZmNF-YC12* was amplified by PCR and the primers are shown in **Supplementary Table S1**. These four cDNA sequences were cloned between SpeI and BamHI sites of the pMDC83-GFP vector. The resulting 35S: ZmNF-YC12-GFP and GFP control vector were transiently expressed in Nicotiana benthamiana leaves through Agrobacterium-mediated infiltration (Chung et al., 2004). Two days later, the fluorescence of the infected leaf tissue was observed under a confocal microscope of Zeiss LSM700 (Zeiss, Jena, Germany).

### 2.9 Transactivation activity in yeast and Gal4/UAS system assay

The CDS (Sequence coding for amino acids in protein) of *ZmNF-YC12* was amplified by PCR with a pair of gene-specific primers (**Supplementary Table S1**), and then ligated to pGBKT7 vector digested by EcoRI/BamHI. A PGBKT7-ZmNF-YC12 construct, pGADT7-T+pGBKT7-Lam (negative control), and pGBKT7-53 plus pGADT7-T (positive control) were transformed into AH109 yeast by lithium acetate-mediated method. The transformed yeast cells were observed on SD/-Trp, SD/Trp-/His/X-a-gal and SD/-Trp/-His/-Ade/X-a-Gal medium for 5 days at 30°C. 35S-ZmNF-YC12 has been shown to contain 35S promoter of the cauliflower-mosaic virus (CaMV), which drives the expression of *ZmNF-YC12*. 35S-Luciferase contains firefly luciferase driven by the constitutive CaMV-35S promoter. The reporter gene constructs (UAS-GUS) and effector constructs (VP16, Gal4, and IAA17) were represented previously by Tiwari (Tiwari et al., 2001). The ZmNF-YC12-GAL4 effector construct contains the full-length *ZmNF-YC12* encoding sequence fused to the N-terminus of the Gal4 DNA-binding domain under the control of CaMV-35S promoter. The 35S-LUC construct was co-transformed as an internal control to standardize the GUS reporter gene expression. β-Glucuronidase (GUS) and luciferase (LUC) enzymatic assays were used to determine in Nicotiana benthamiana leaves and protoplasts prepared from the cells after 5 d of subculture. Cell walls were digested at room temperature for 2 h in a solution containing 0.1% (w/v) pectinase (Sigma-Aldrich), 0.5% (w/v) Macerozyme RS (Serva),1% (w/v) cellulase Onozuka R-10 (Serva) and 0.25 M mannitol. The isolated protoplasts were transformed with either mock DNAs or 20 μg of the reporter and effector constructs using PEG method. LUC substrate (Promega, Madison, WI, USA) was prepared on the basis of the manufacturer’s instructions. Ten microliters of sample extract was mixed with 50 μl of substrate, and the luciferase activity was determined on a Zylux FB15 luminometer (Fisher Scientific, Pittsburgh, PA, USA). GUS activities were measured by fluorometry method with 4-methylumbelliferyl glucuronide as the substrate. The relative reporter gene activity was described as the ratio of LUC to luciferase activities, expressed in units of 4-methylumbelliferyl fluorescence per 40 μl of extract per hour and photons per 10 μl of extract per minute, respectively (Gampala et al., 2001). The data represent the mean ± SD from three biological replicates.

### 2.10 Maize transformed plants drought resistance and resilience analysis

The specific fragments of 180 bp in the ORF region of *ZmNF-YC12* were selected to design primers (**Supplementary Table S1**). Virus-induced gene silencing (VIGS) was used to silence *ZmNF-YC12*. BMV-GFP is the vector for transiently silencing gene expression. The *ZmNF-YC12* gene was isolated from Va35 maize and loaded into the BMV-GFP vector to obtain the ZmNF-YC12-BMV fusion. Subsequently, BMV-ZmNF-YC12 and empty BMV-GFP were introduced into Agrobacterium tumefactions strain GV3101. As described previously, the Agrobacterium cultures of BMV-ZmNF-YC12 and empty BMV-GFP were mechanically inoculated into the leaves of A. tumefaciens-infiltrated N. benthamiana plants at 24 h post-infiltration (Chen et al., 2017). Tobacco leaves were harvested for extraction of virus according to a previous method after three days (Chen et al., 2017). The extracted BMV-ZmNF-YC12 and BMV-GFP tobacco virus were infected into the second leaf of Va35 maize and cultured in a 20°C light incubator with a light-transmitting plastic cover for six days. The plastic cover was then removed and placed in a 22°C lamp incubator. BMV-GFP serves as a wild-type control, hereinafter referred to as BMV. The expressions of *ZmNF-YC12* were detected 7 days after virus infection on *ZmNF-YC12* mutant (BMV-YC12-1~5) and BMV (**Supplementary Figure S1**). Among them, BMV-YC12-1 and BMV-YC12-5 mutants had higher silence rate. The two strains were selected for drought stress, and the soil RWC was kept at 45% (moderate drought stress) for 5 days, followed by watering (the soil RWC at 95%) for 3 days. The containers were weighed after being watered to saturation (initial weight). The formula for calculating soil RWC was (fresh weight-dry weight)/(initial weight-dry weight) ×100.

### 2.11 Measurement of the relative water content of leaves, the malondialdehyde content, chlorophyll content, photosynthetic rate, leaf relative water content, and water loss rate

Chlorophyll content was determined according to Lakra et al. (2015). Net photosynthetic rate was measured by an open infrared gas analysis system (Li-COR6400, Lincoln, NE, USA). The extraction of malondialdehyde (MDA) was determined by thiobarbituric acid reaction. The relative water content of leaves (RWC) was measured by weighing method, and the water loss rate of each strain was calculated within 5 h.

### 2.12 Measurement of superoxide dismutase, peroxidase, catalase, and ascorbate peroxidase activity

Took maize seedlings with consistent growth for enzyme activity measurement and performed three biological replicates. The activities of peroxidase (POD) and superoxide dismutase (SOD) were determined according to Azevedo et al. (1998). Ascorbate peroxidase (APX) and catalase (CAT) were extracted with 0.05 M phosphate buffer (Ph=7.5) containing 1% PVP (Kaur et al., 2009). Both the homogenates were centrifuged at 10,000 ×g for 20 min, and the supernatant was used for the determination of the above enzymes. All enzymes were assayed at 30°C. The components of enzyme assay system were preincubated at 30°C for 20 min before starting the reaction, except for the enzyme. Each sample was tested thrice at 4°C.

### 2.13 Measurement of soluble protein, proline content, pyrroline-5-carboxylic acid synthetase activity, proline dehydrogenase activity

Soluble protein (Sp) content in leaves was measured by Coomassie brilliant blue staining. The content of free proline (Pro) was measured by sulfosalicylic acid extraction-ninhydrin chromogenic method. The Pyrroline-5-carboxylic Acid Synthetase Activity (P5CS) was determined according to Yang et al. (2009). The activity of proline dehydrogenase (ProDH) was measured according to Esteban et al. (2001).

## 3 Results

### 3.1 Identification and characterization of ZmNF-Y family genes in maize

Maize family members containing NF-Y domains were analyzed using SMART, CDD, and PFAM. After removing different transcripts of the same gene, a total of 52 non-redundant protein sequences (16 ZmNF-YAs, 19 ZmNF-YBs, and 17 ZmNF-YCs) representing the primary transcript were identified (**Supplementary Table S3**). All 52 ZmNF-Ys contained one highly conserved domain, which is divided into three NF-Y subfamilies that were randomly and unevenly distributed on the 10 maize chromosomes. To distinguish these newly identified genes, we renamed these genes based on their subfamily branches (*ZmNF-YA1-ZmNF-YA16, ZmNF-YB1-ZmNF-YB19, and ZmNF-YC1-ZmNF-YC17*). The protein lengths of the 52 ZmNF-Ys ranged from 90 AA (ZmNF-YA3) to 830 AA (ZmNF-YB19) (**Table S3**), showing a wide ZmNF-Y length distribution. The predicted molecular weights ranged from 10394.6 Da (ZmNF-YB16) to 92076.2 Da (ZmNF-YB19), and the predicted theoretical isoelectric points (pI) ranged from 4.1706 (ZmNF-YB15) to 12.3854 (ZmNF-YA3).

### 3.2 Phylogenetic, gene structure, and motif analyses of ZmNF-Y family genes

**Figure 1:**
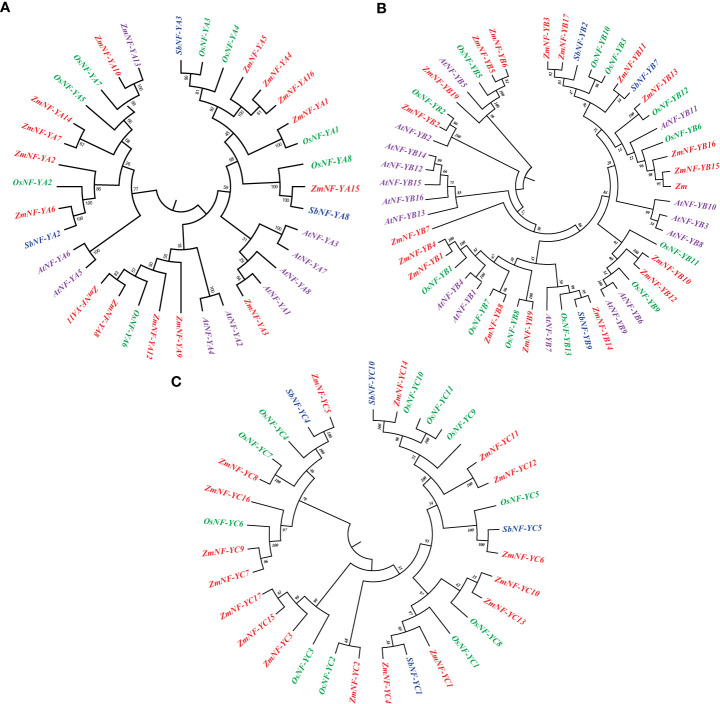
Phylogenetic classification of NF-Ys from maize, Arabidopsis, sorghum, and rice. **(A)** Subfamily A of NF-Y. **(B)** Subfamily B of NF-Y. **(C)** Subfamily C of NF-Y. Red, green, blue, and purple represent maize, rice, sorghum, and Arabidopsis genes, respectively.

To study the evolutionary relationship of NF-Y genes, 24, 28, 31, and 52 NF-Y genes were selected from Arabidopsis, sorghum, rice, and maize, respectively, and an evolutionary tree was constructed using MEGA 7.0. As shown in **Figure 1**, the NF-Y gene is divided into three subfamilies. In the NF-YA subfamily, *ZmNF-YA3* and *AtNF-YA1* are located in the same branch and are thus homologous proteins. In the NF-YB subfamily, *OsNF-YB2*, *ZmNF-YB2*, and *AtNF-YB2* are located in the same branch, indicating their similarities. In addition, (*ZmNF-FC8* and *OsNF-YC7*) and (*ZmNF-YC4* and *ZmNF-YC5*) in the NF-YC subfamily are homologous genes, respectively. The high homology of NF-Y genes in different species suggests that they may have similar biological functions. This evolutionary feature also indicates that there may be genetic redundancy and functional differentiation in this gene family.

**Figure 2:**
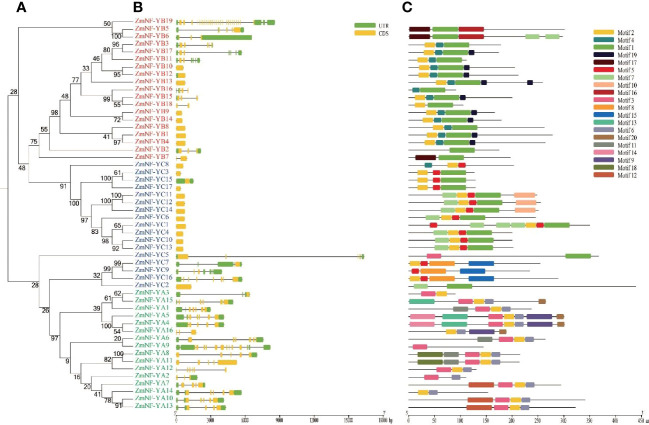
Phylogeny, gene structure, and distribution of conserved motifs in ZmNF-Y proteins. **(A)** The phylogenetic tree was built based on the full amino acid sequences of 52 ZmNF-Y proteins using the neighbor-joining method implemented by MEGA software. **(B)** Gene structures of the 52 ZmNF-Y genes. Exons and introns are indicated by orange rectangles and thin strains, respectively, and untranslated regions (UTRs) are indicated by green rectangles. **(C)** Motifs of the ZmNF-Y proteins were identified using the online MEME program; Rectangles with different colors represent different protein motifs., and motif details are listed in **Supplementary Table S2**.

To better understand the diversity of ZmNF-Y members, gene structure and motif analysis were generated (**Figure 2**). All ZmNF-YA genes in the NF-YA except *ZmNF-YA11*, *ZmNF-YA12*, and *ZmNF-YA16* contained UTR structures. In the NF-YB subfamily, *ZmNF-YB19* contained the largest number of exons (26), and other ZmNF-YB genes contained between 1 and 5 exons, (*ZmNF-YB10/12/1/4* contained only one exon). In the ZmNF-YC subfamily, 13 ZmNF-YCs contained one exon. Although *ZmNF-YC5* contained only 5 exons, it had the longest gene length (16800 bp). The motif analysis showed that *ZmNF-YB19* contained no motifs, but it has the largest number of exons and the largest protein molecular weight in ZmNY-F family, maybe its biological function may thus be significantly different from that of other genes. ZmNF-YB genes all contained motif 1. In NF-YC, all NF-YC proteins except ZmNF-YC2/5 contained motif 5. The gene structure, number, and arrangement of motifs on adjacent evolutionary tree branches were similar, which suggested that they may have the same biological function.

### 3.3 Collinearity analysis between ZmNF-Y and AtNF-Y, OsNF-Y, SbNF-Y

**Figure 3:**
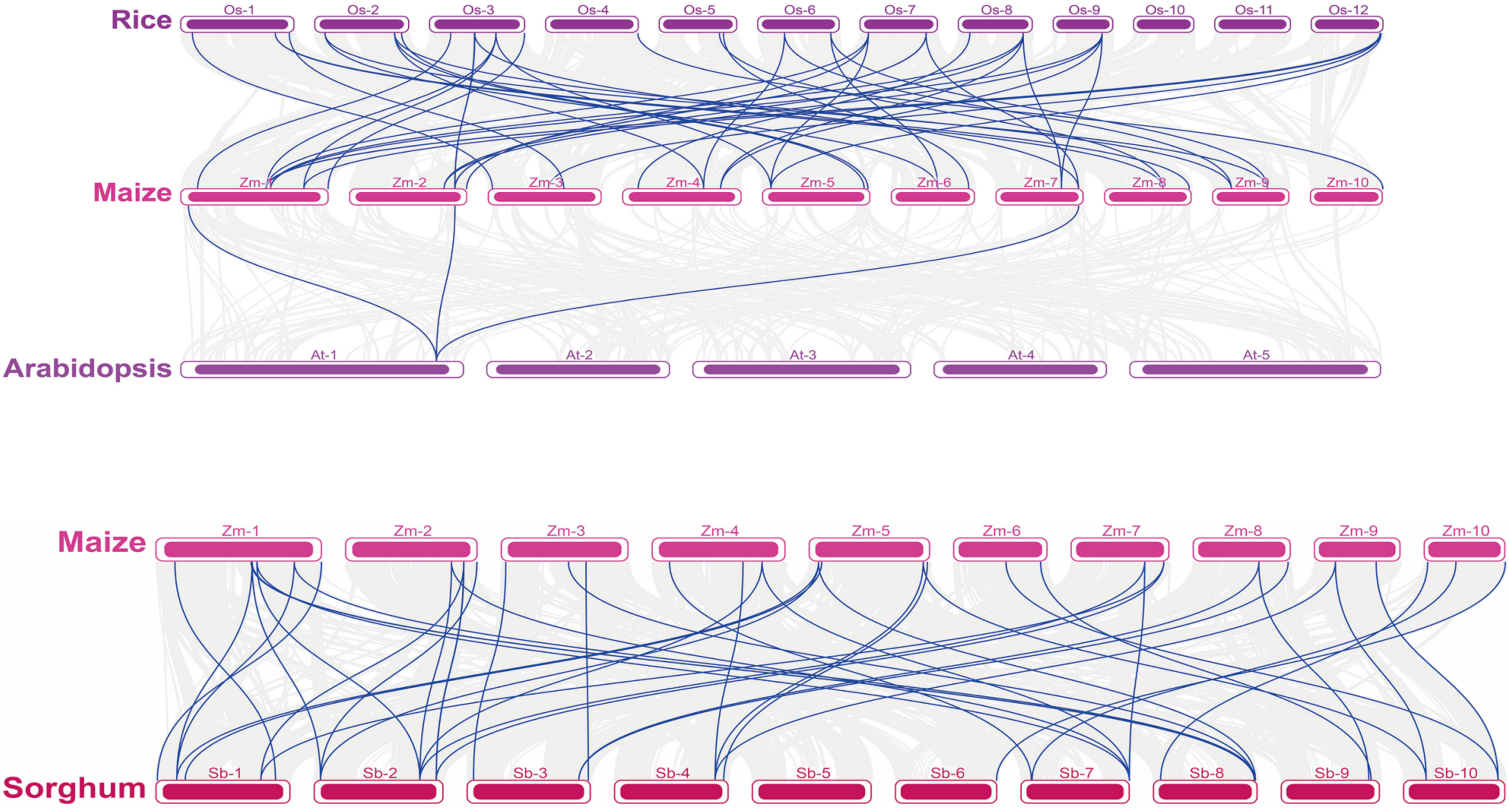
Collinearity analysis of NF-Y family genes in different species. At, *Arabidopsis thaliana*; Os, rice; Sb, sorghum; Zm, maize. The number on the horizontal line in each species represents the chromosome to which it belongs.

A collinearity analysis is an important tool for analyzing species evolution. To analyze the similarities and differences between the ZmNF-Y and NF-Y genes in other species, we conducted a collinearity analysis of four species of maize, rice, Arabidopsis, and sorghum and focused on the NF-Y gene display. As shown in **Figure 3**, maize and rice, Arabidopsis, and sorghum all showed replication relationships in the NF-Y gene, but there was a lower collinearity relationship between the dicotyledon Arabidopsis and maize, (only three gene pairs). In addition, *AtNF-YA3* showed a collinearity relationship with the three NF-Y genes (*ZmNF-YA1/5/11)* of maize. There were 27 and 30 NF-Y gene pairs between maize and rice and sorghum, respectively, and there was a collinearity relationship between a sorghum/rice NF-Y gene and multiple maize NF-Y genes. Of these, the NF-YA gene (*SbNF-YA7*) in sorghum showed a gene replication relationship with NF-YA genes (*ZmNF-YA10/13/14*) in maize, and there was a replication relationship between the *OsNF-YB1* gene of rice and the *ZmNF-YB1/4/8* genes of maize. This suggests that the NF-Y gene is strongly conserved in the evolutionary process, and the evolutionary process of maize is more complex and rigorous than sorghum and rice.

### 3.4 Expression patterns of ZmNF-Y genes in different tissues and in response to water stress and rewatering

Gene expression is regulated by the cis acting element upstream of ATG. The cis-acting elements in the *ZmNF-Y* genes at 2000 bp upstream of ATG were predicted to analyze factors regulating the expression of NF-Y genes (**Supplementary Figure S2**). The main targets of analysis were the cis-acting elements of abiotic stress, such as defense- and stress-responsive elements (TC-rich repeats), low temperature-responsive elements (LTR), drought-inducible MYB binding site elements (MBS), abscisic acid (ABA) -responsive elements (ABREs), MeJA-responsive elements (CGTCA-motifs), and salicylic acid reaction (TCA-elements) elements, and cis-acting regulatory element essential for the anaerobic induction. ABA signaling plays an important role in drought response pathways Of the NF-Y genes, 36/52 (69.23%) contained ABRE cis-acting elements (including 7 *ZmNF-A*, 11 *ZmNF-B*, and 17 *ZmNF-YC*), and 12 contained MBS cis-acting elements (including 6 *ZmNF-YB* genes and 6 *ZmNF-YC* genes). None of the ZmNF-YA genes contained MBS cis-acting elements. This cis-acting element analysis showed that NF-Y gene may play a role in coping with abiotic stress and plant growth and development, and *ZmNF-YB* and *ZmNF-YC* may play a more important role in participating in drought stress response.

**Figure 4:**
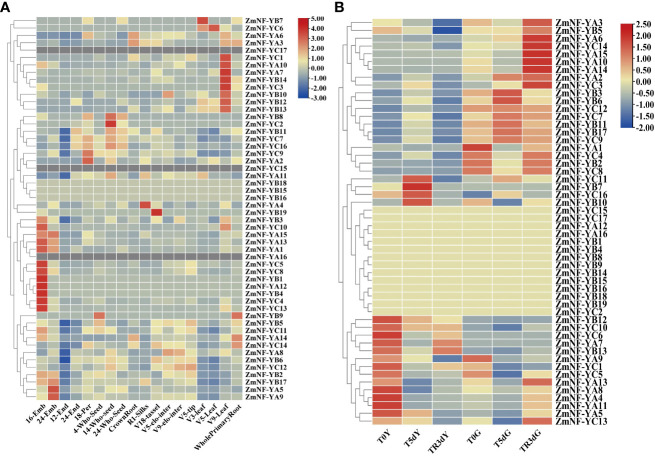
Expression patterns of 52 ZmNF-Y genes in various maize organs and under different stress conditions. **(A)** The expression profiles of ZmNF-Y genes in 18 different maize organs or tissues; **(B)** Expression profiles are transcriptome data of maize seedlings under normal, drought, and rewatering conditions. In the figure, T0Y, T5dY, TR3dY, and T0G, T5dG, TR3dG, “Y” represents the leaves samples, “G” represents the root samples, “T0, T5d, and TR3d” represent pre-drought stress, 5 d after drought stress, and 3 d after rewatering, respectively. The heat map was generated based on the FPKM (fragments per kilobase of exon model per million mapped fragments) values that were transformed to log2 (value +1). Red and green blue gradients indicate an increase or decrease, respectively.

To explore the biological functions of the NF-Y gene family, the transcriptome data of maize were used to investigate the expression profiles of the ZmNF-Y genes at different stages, organs, and tissues, including 16DAP_Embryo, 24DAP_Embryo, 12DAP_Endopsperm, 24DAP_Endosperm, 18DAP_Pericarp, 4DAP_Whole_Seed, 14DAP_Whole_seed, 24DAP_Whole_Seed, CrownRoot_V7, R1_Silks, V18_tassel, V5_elonagetd_internode, V9_elongated_internode, V5_Shoot_tip, V3_Leaf, V5_Leaf, V9_Leaf, WholePrimary, and Root_7d (**Figure 4A**). The results indicated that most ZmNF-Y genes were extensively expressed in many organs and tissues, except 12DAP_endosperm. Of these, *ZmNF-YA5*, *ZmNF-YB11*, *ZmNF-YC7*, and *ZmNF-YC9* were expressed at high levels in the embryo, endosperm, and whole seed; and *ZmNF-YB2*, *ZmNF-YB11*, *ZmNF-YC7*, and *ZmNF-YC9* were expressed at high levels in the roots, stems, leaves, internode, silks, and tassels. However, the expressions of *ZmNF-YA12*, *ZmNF-YB7*, *ZmNF-YB9*, *ZmNF-YB19*, and *ZmNF-YC3* were only detected in individual tissues, and the FPKM value was less than 1. Although *ZmNF-YB15*, *ZmNF-YB16*, and *ZmNF-YB18* were detected in high-throughput sequencing, their expression levels tended to zero, while *ZmNF-YA16*, *ZmNF-YC15*, and *ZmNF-YC17* were not detected. Various *ZmNF-Y* genes have different expression patterns, and this result shows that the function of the ZmNF-Y gene has been differentiated in the long evolutionary process.

Transcriptome data were used to detect the expression levels of ZmNF-Ys in drought and rewatering treatments (**Figure 4B**). *ZmNF-YB3, ZmNF-YB7*, *ZmNF-YB11*, *ZmNF-YB17*, *ZmNF-YC3*, *ZmNF-YC9*, and *ZmNF-YC12* had similar expression patterns in response to drought and rewatering: the expression was significantly up-regulated after stress in leaves and significantly down-regulated after rewatering. *ZmNF-YA8* and *ZmNF-YC6* also showed similar expression patterns: the expression was significantly down-regulated after stress in leaves and significantly up-regulated after rewatering; *ZmNF-YA13* was significantly down-regulated after stress in roots and leaves and up-regulated after rewatering; and *ZmNF-YA14* was significantly down-regulated in roots and up-regulated after rewatering. These genes thus play an important role in drought and rewatering treatments.

### 3.5 Important candidate ZmNF-Y genes under drought and rewatering treatments

**Figure 5:**
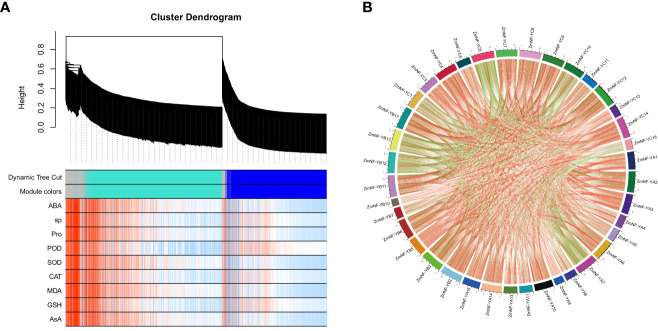
Weighted correlation network analysis (WGCNA) coexpression network and module-trait correlation analysis under drought and rewatering treatment. **(A)** Hierarchical cluster tree showing coexpression modules: different colors indicate different modules. Correlation analysis between different modules and physiological and biochemical indices during drought and rewatering. **(B)** Lines in the figure represent Pearson correlation information about gene expression values. Red and green represent positive and negative correlations, respectively. The darker the color or the thicker the lines, the higher the correlation intensity.

To further identify candidate differentially expressed *ZmNF-Y* genes under drought and rewatering treatments, a WGCNA was conducted in this study. A correlation analysis was conducted between the gene co-expression modules identified by the WGCNA and the nine physiological and biochemical indicators related to drought resistance (**Figure 5A**; **Supplementary Figure S3**). The results showed that the blue and turquoise modules were significantly correlated with these indicators. In these important modules, *ZmNF-YA8* and *ZmNF-YC6* were found to respond to ABA in the turquoise module, *ZmNF-YB11* responded to Pro in the turquoise module, *ZmNF-YB3* responded to POD in the blue module, and *ZmNF-YC12* and *ZmNF-YC3* responded to SOD located in the blue module. It is thus speculated that these genes play important roles in drought and rewatering treatments.

The correlation coefficients of the expression levels in the transcriptome of the 52 genes were further calculated, and Cytoscape software was used to conduct a co-expression network analysis (**Figure 5B**; **Supplementary Table S4**). The results showed that *ZmNF-YC3, ZmNF-YC12, ZmNF-YC6, ZmNF-YA2*, *ZmNF-YB17*, *ZmNF-YB11*, *ZmNF-YC4*, *ZmNF-YC7*, *ZmNF-YC8*, *ZmNF-YC14*, and *ZmNF-YB3* had high degrees and k-core values, which belong to the core node gene.

### 3.6 Abiotic stress-related response of *ZmNF-Y* Genes

**Figure 6:**
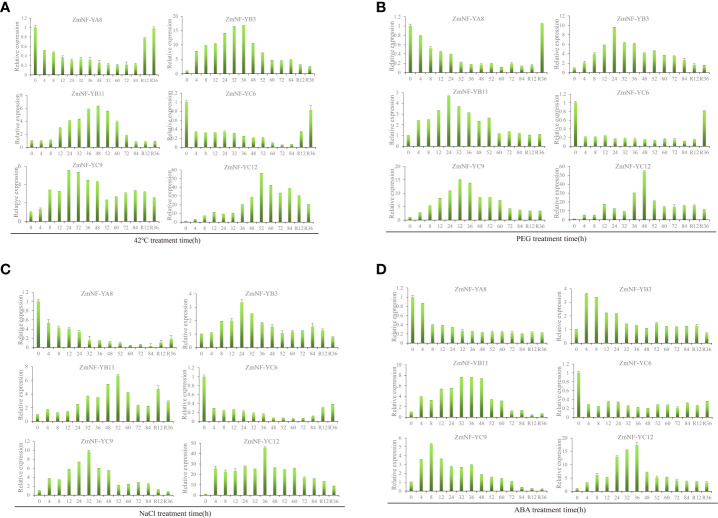
Expression patterns of ZmNF-Y genes in response to 42°C, PEG, NaCl, and ABA treatments. The relative expression levels of six ZmNF-Y genes were examined by qRT-PCR. **(A)** Relative expressions of six ZmNF-Y genes under 42°C treatment at 0, 4, 8, 12, 24, 36, 48, 52, 60, 72, 84 h, and R12, R36 h; **(B)** Relative expressions of 12 ZmNF-Y genes under 20% PEG6000 treatment; **(C)** Relative expressions of six ZmNF-Y genes under 200 mmol/L NaCl treatment; **(D)** Relative expressions of six *ZmNF-Y* under 5 µmol/L ABA treatment. The error bars represent standard deviations (SD); y-axes are scales of relative expression levels; x-axes show the time course of the treatment for each condition.

An analysis of the expression difference, the correlation between genes and the physiological and biochemical indexes, and the co-expression network analysis identified six core drought resistance genes (*ZmNF-YA8*/*YB3*/*YB11*/*YC6*/*YC9*/*YC12*). The expression patterns of these genes in response to various abiotic stresses were then further analyzed. Under a high temperature treatment at 42 °C, the expression of *ZmNF-YA8* continuously decreased to 84 h, but it was relieved and recovered to the pre-treatment level 36 h later. *ZmNF-YB3* reached its highest level at 36 h and then continued to decrease following stress relief. The trend in variation of *ZmNF-YB11* was similar to that of *ZmNF-YB3*, except that the expression of *ZmNF-YB11* was highest at 48 h under high temperature stress, and following stress relief, the expression level was consistent with that of 84 h. The expression of *ZmNF-YC6* decreased to its lowest level in 72 h under stress. After stress was relieved, the expression levels gradually increased. The expression level of *ZmNF-YC9* was the highest at 24 h, and that of *ZmNF-YC12* reached its peak at 52 h after stress, at which time the expression level was 56.13 times that of when untreated, and it gradually decreased at subsequent time points (**Figure 6A**).

Under PEG stress, the expression of *ZmNF-YA8* decreased gradually, but it increased sharply to pre-treatment levels 36 h after rewatering. The expression level of *ZmNF-YB3* was the highest at 24 h, but it slowly decreased after rewatering. The expression level of *ZmNF-YB11* peaked at 24 h and remained stable after 60 h, while that of *ZmNF-YC6* remained below 0.3 during the treatment but increased suddenly at 36 h after rewatering. ZmNF-YC9 showed a similar trend to *ZmNF-YB11*, and the expression level of *ZmNF-YC9* was the highest at 32 h. The expression level of *ZmNF-YC12* reached its peak at 48 h (by 54.27 times) but gradually decreased after rewatering (**Figure 6B**).

Under NaCl treatment, the expression of *ZmNF-YA8* decreased to its lowest level at 84 h, but after stress relief, the level slowly increased. The expression level of *ZmNF-YB3* was the highest at 24 h, but it gradually decreased when stress was relieved. The expression of *ZmNF-YB11* first increased and then decreased under stress, and the highest level was reached at 52 h. The variation trend of *ZmNF-YC6* was similar to that of *ZmNF-YA8*, and the expression level of *ZmNF-YC6* was the lowest at 60 h. The expression of *ZmNF-YC9* peaked at 32 h; it slowly decreased to the untreated level after stress was relieved. That of *ZmNF-YC12* reached a peak at 36 h (45.84 times that before treatment), and slowly decreased after stress was relieved (**Figure 6C**).

Under ABA treatment, the expression of *ZmNF-YA8* suddenly decreased at 8 h, and then slowly decreased to its lowest level at 84 h. *ZmNF-YB3* increased sharply at 4 h after stress and then gradually decreased; it subsequently continued to decrease after stress was removed. The expression level of *ZmNF-YB11* was the highest at 32–48 h, but it increased slowly following stress relief, while that of *ZmNF-YC6* remained at 0.20–0.35 after stress and was consistently at a low level. The expression level of ZmNF-YC9 was the highest at 8 h, and that of *ZmNF-YC12* reached a peak at 36 h but decreased after stress was relieved (**Figure 6D**).

In summary, *ZmNF-YA8*/*YB3*/*YB11*/*YC6*/*YC9*/*YC12* showed different expression patterns under a high temperature of 42 °C, PEG-induced drought, and NaCl and ABA treatments. After treatment, the expressions of *ZmNF-YB3*, *ZmNF-YB1*1, *ZmNF-YC9*, and *ZmNF-YC12* first increased then decreased, and the difference in the expression of *ZmNF-YC12* was the largest. After normal growth conditions were restored, the expressions of these four genes showed an increased, decreased, or unchanged trend. After treatment, *ZmNF-YA8* and *ZmNF-YC6* were induced to downregulate the expression, and after normal growth conditions were restored, the expression levels rose slowly. The results showed that ZmNF-Ys play important roles in stress and recovery compensation, but the mechanisms of their actions differ.

### 3.7 ZmNF-Y is located in the nucleus and acts as a transcription activator

**Figure 7:**
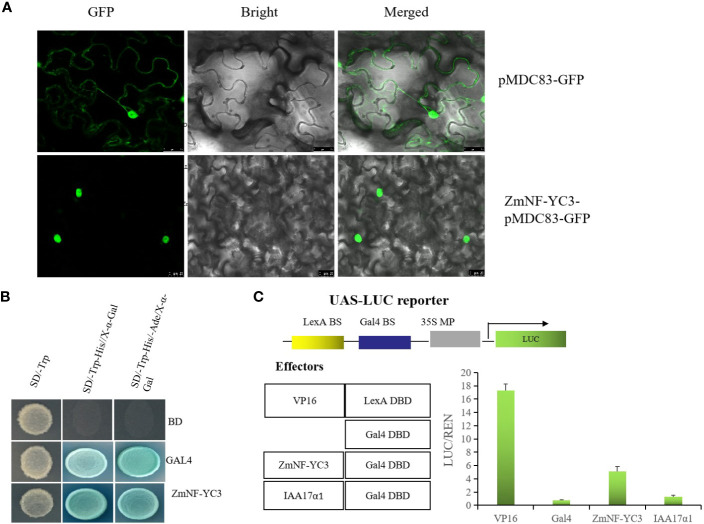
ZmNF-YC12 subcellular localization, yeast trans activation system, and Gal4-LexA/UAS system. **(A)** Fusion proteins were transiently expressed under the control of CaMV35S promoter in tobacco leaves and were subsequently observed under a laser scanning confocal microscope. Bars = 25 µm. **(B)** The negative and positive controls were pGADT7-T+pGBKT7-Lam (BD) and pGADT7-T+pGBKT7-53 (GAL4), respectively. **(C)** The reporter factor (LUC) and internal reference (REN) were expressed by the Mini CaMV35s promoter and had binding sites of yeast GAL4 and LexA (GAL4-BS and LexA-BS). The binding domains of the effectors were GAL4 and LexA, respectively, and the transcription factor coding sequences were fused with only one binding domain: LexA DBD-VP16, GAL4 DBD-GBF1, GAL4 DBD-IAA17α1, or GAL4 DBD. The expression values of the co-transformation between different effectors and reporters are shown.

Based on the above results it is concluded that ZmNF-YC12 play a crucial role in drought tolerance and recovery in maize and therefore we selected *ZmNF-YC12* to further explore its function and mechanism of action. The fusion expression vector ZmNF-YC12-pMDC83-GFP was transferred into tobacco to achieve an transient expression, and laser confocal microscope observation revealed that ZmNF-YC12-pMDC83-GFP only emitted green fluorescence in the nucleus (**Figure 7A**). This indicates that the ZmNF-YC12 protein is localized in the nucleus and is therefore predominantly a nuclear protein. To analyze the activity of ZmNF-YC12, we first analyzed the transcriptional activation of the ZmNF-YC12-encoded protein in yeast and found that pGBKT7-ZmNF-YC12 had the same activity as the positive control in yeast; this indicates that the ZmNF-YC12 protein has transcriptional activation activity (**Figure 7B**). We also analyzed whether *ZmNF-YC12* is an activator or an inhibitor in the Gal4-LexA/UAS system. When the reporter gene was co-transformed into protoplasts with a LexAVP16 fusion structure, the LexA DBD-VP16 fusion protein with a LexA DNA binding domain was highly expressed. The expression value of the GAL4 DBD-ZmNF-YC12 fusion protein with a GAL4 binding domain was significantly higher than that of the transcription activator IAA17α1 and the empty vector GAL4. These results indicate that *ZmNF-YC12* is a transcriptional activator (**Figure 7C**).

### 3.8 Silencing of ZmNF-YC12

#### 3.8.1 Silencing of ZmNF-YC12 increases water loss and reduces drought resistance and recovery ability of maize

**Figure 8:**
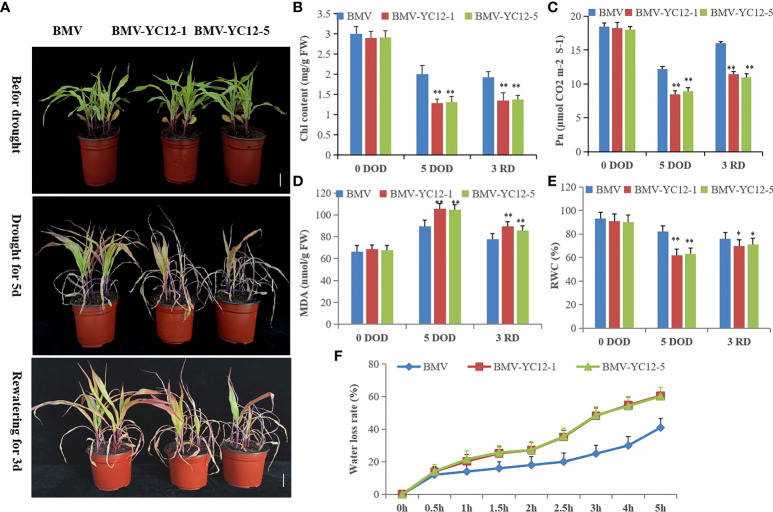
Analysis of drought resistance and recoverability when silencing ZmNF-YC12. **(A)** Leaf wilting and death of BMV and ZmNF-YC12 mutants under drought stress and rewatering; **(B)** Chlorophyll content of BMV and ZmNF-YC12 mutants; **(C)** Net photosynthetic rate of BMV and ZmNF-YC12 mutants; **(D)** MDA contents of BMV and ZmNF-YC12 mutants; **(E)** Relative water contents of BMV and ZmNF-YC12 mutants; **(F)** Water loss rates of BMV and ZmNF-YC12 mutants. Mean values and standard errors (bar) are shown from three independent experiments. Independent t-tests for equality of means demonstrated that there were significant differences between the control and treatments in BMV and ZmNF-YC12 mutants (*P value < 0.05). The figures below are the same. The experiment was repeated at least three times independently and the data are shown as mean ± SD. Significant differences (*P < 0.05 and **P < 0.01) are based on Student’s t-tests.

Virus-induced gene silencing (VIGS) was used to silence *ZmNF-YC12* and study the drought resistance and recovery ability of maize. Five silent strains were obtained by virus inoculation (**Supplementary Figure S1**), and two strains with high silencing efficiency (BMV-YC12-1 and BMV-YC12-5) were selected to conduct a functional analysis. BMV-YC12-1, BMV-YC12-5, and BMV were then subjected to drought stress for five and three days, respectively. The leaves of BMV-YC12-1, BMV-YC12-5, and BMV curled up, wilted, and lost their green color, and the leaf tips gradually withered. However, the growth of BMV seedlings was superior to that of BMV-YC12-1 and BMV-YC12-5 seedlings. Following three days of rewatering, BMV resumed growth, but the growth of BMV-YC12-1 and BMV-YC12-5 did not return to normal levels (**Figure 8A**). The chlorophyll content of BMV leaves was significantly higher than that of BMV-YC12-1 and BMV-YC12-5 leaves after drought stress and rewatering (**Figure 8B**). Following drought stress, the net photosynthetic rate of BMV-YC12-1, BMV-YC12-5, and BMV decreased, but that of BMV was significantly higher than BMV-YC12-1 and BMV-YC12-5. After rewatering, the rates of all increased, but the recovery of BMV was higher (**Figure 8C**). The MDA content is an important physiological index used to evaluate damage to the leaf cell membrane under drought stress. Prior to treatment, there was no significant difference between the two indexes among the strains, but after five days of drought stress, the contents of BMV-YC12-1 and BMV-YC12-5 were significantly higher than those of BMV. After three days of rewatering, the MDA content of each strain decreased, but that of BMV-YC12-1 and BMV-YC12-5 was still higher than that of BMV (**Figure 8D**). These results show that by silencing *ZmBF-YC12*, the chloroplast of the plant was seriously damaged, the photosynthetic rate was significantly reduced, and the cell membrane was severely damaged.

The RWC can reflect the water potential of plant leaves and is an important indicator of plant drought resistance. Plants that can maintain high RWC values under water stress have strong drought resistance. After drought stress, the RWC of the leaves of all strains decreased, but the leaf water content of BMV was significantly higher than that of BMV-YC12-1 and BMV-YC12-5. After rewatering, the RWC recovered, but that of BMV remained higher than that of BMV-YC12-1 and BMV-YC12-5 (**Figure 8E**). The rate of water loss from leaves was further measured (**Figure 8F**); greater water loss was seen in BMV-YC12-1 and BMV-YC12-5 compared to BMV plants. These results indicate that silencing *ZmBF-YC12* does indeed reduce the drought resistance and recovery ability of maize.

#### 3.8.2 Silencing ZmNF-YC12 reduces the activity of antioxidant enzymes in maize under drought and rewatering treatments

**Figure 9:**
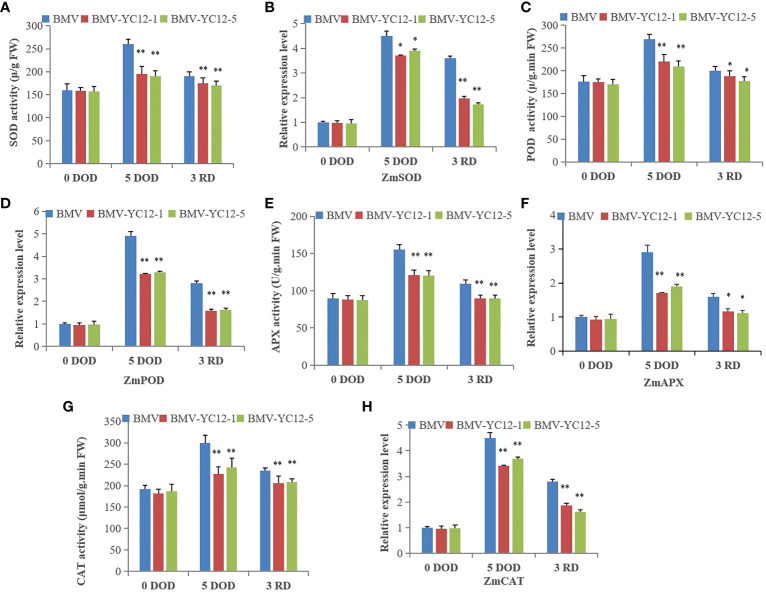
Analysis of effects of drought and rewatering treatments on antioxidant enzyme activity and gene expression in leaves of different maize strains. **(A)** Superoxide dismutase activity of BMV and ZmNF-YC12 mutants under drought stress and rewatering; **(B)** Expression level of *ZmSOD* in BMV and ZmNF-YC12 mutants under drought and rewatering treatments; **(C)** Antioxidant enzyme activity of BMV and ZmNF-YC12 mutants; **(D)** Expression level of *ZmPOD* in BMV and ZmNF-YC12 mutants; **(E)** Ascorbate peroxidase activity of BMV and ZmNF-YC12 mutants; **(F)** Expression level of *ZmAPX* in BMV and ZmNF-YC12 mutants; **(G)** Catalase activity of BMV and ZmNF-YC12 mutants; **(H)** Expression level of *ZmCAT* in BMV and ZmNF-YC12 mutants. The experiment was repeated at least three times independently and the data are shown as mean ± SD. Significant differences (*P < 0.05 and **P < 0.01) are based on Student’s t-tests.

After drought stress, the MDA of maize strains increased significantly (**Figure 6D**), which indicated that drought stress damaged the maize cells. At this time, a series of antioxidant enzymes (SOD, POD, CAT and APX) played scavenging roles and maintained cell functions and metabolic activities. There were no significant differences between the SOD, POD, CAT, and APX enzyme activities among the strains before stress treatment. After five days of stress, the enzyme activities of BMV-YC12-1 and BMV-YC12-5 were lower than those of WT, and they remained lower than WT after rewatering. The expression analysis of four antioxidant enzyme genes (*ZmSOD, ZmpOD, ZmAPX*, and *ZmCAT*) showed that their change trends were consistent with their corresponding antioxidant enzyme activities (**Figures 9A–H**). These results indicate that silencing *ZmNF-YC12* reduces the activity of antioxidant enzymes and reduces the drought resistance and recovery ability of maize.

#### 3.8.3 Silencing of *ZmNF-YC12* reduces content of osmolytes in maize under drought and rewatering treatments

**Figure 10:**
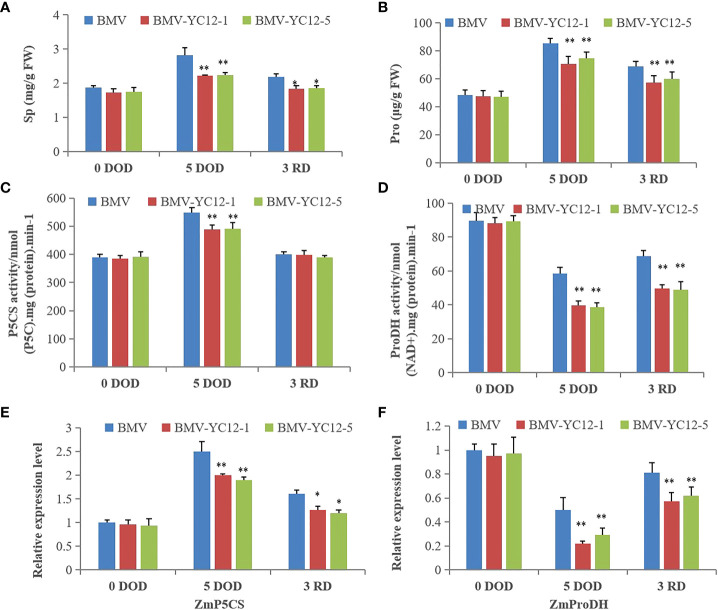
Analysis of the effects of drought and rewatering treatments on the osmoregulation substance and gene expressions in leaves of different maize strains. **(A)** Soluble protein content of BMV and ZmNF-YC12 mutants; **(B)** Proline content of BMV and ZmNF-YC12 mutants; **(C)** Pyrroline-5-carboxylic acid (P5CS) synthetase activity of BMV and ZmNF-YC12 mutants; **(D)** Proline dehydrogenase (ProDH) activity of BMV and ZmNF-YC12 mutants; **(E)** Expression level of *ZmP5CS* in BMV and ZmNF-YC12 mutants; **(F)** Expression level of *ZmProDH* in BMV and ZmNF-YC12 mutants. The experiment was repeated at least three times independently and the data are shown as mean ± SD. Significant differences (*P < 0.05 and **P < 0.01) are based on Student’s t-tests.

Osmolytes, such as soluble protein and proline, participate in regulating the osmotic potential of plants. In this respect, they maintain the balance and stability of cell turgor, and enable various metabolic processes in the plant body to function normally. As shown in **Figures 10A, B**, there were no differences in the soluble protein and proline contents of each strain prior to stress. Although they all increased after stress, the contents of BMV were higher than those of BMV-YC12-1 and BMV-YC12-5. After rewatering, the contents in BMV decreased but remained higher than those of BMV-YC12-1 and BMV-YC12-5. Pyrroline-5-carboxylic acid (P5CS) is a key enzyme involved in proline synthesis, and proline dehydrogenase (ProDH) is a key enzyme in mitochondria that catalyzes the degradation of proline. The activities of P5CS and ProDH were increased and decreased, respectively, after stress in all strains, but the reverse trend was seen following rewatering (**Figures 10C, D**), and this trend was the same as that of the proline content. A further analysis of the expressions of *ZmP5CS* and *ZmProDH* (**Figures 10E, F**) showed that the change trend was consistent with the corresponding enzyme activity. These results indicate that *ZmNF-YC12* may be involved in regulating the synthesis or degradation of soluble sugar and proline in maize leaves. After drought stress treatment, silencing *ZmNF-YC12* reduced the accumulation of osmoregulation substances in cells, improved the osmotic potential in maize, and made maize sensitive to drought.

#### 3.8.4 Silencing of *ZmNF-YC12* reduces the expression of genes responding to drought stress under drought and rewatering treatments

**Figure 11:**
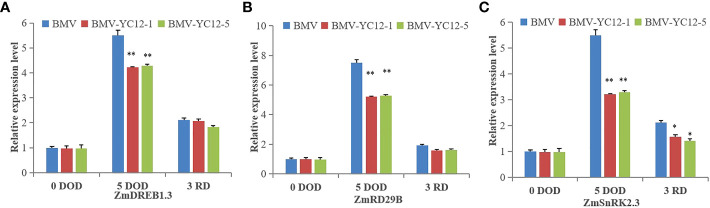
Analysis of the expression of drought stress responsive genes in different maize strains under drought and rewatering treatments. **(A)** Expression level of dehydration-responsive element-binding protein gene (*ZmDREB1.3*) in BMV and ZmNF-YC12 mutants; **(B)** Expression level of dehydration-responsive protein 29B gene (*ZmRD29B*) in BMV and ZmNF-YC12 mutants; **(C)** Expression level of sucrose non-fermenting-1-related protein kinase 2.3 gene (*ZmSnRK2.3*) in BMV and ZmNF-YC12 mutants. The experiment was repeated at least three times independently and the data are shown as mean ± SD. Significant differences (*P < 0.05 and **P < 0.01) are based on Student’s t-tests.

The expressions of three genes that positively responded to drought stress in each strain under the drought stress and rewatering treatments were analyzed (**Figures 11A–C**). Under drought stress, there was a greater increase in the expressions of these three genes in BMV compared to BMV-YC12-1 and BMV-YC12-5; after watering was resumed, the expression of the three genes remained higher in BMV. These results showed that silencing *ZmNF-YC12* indirectly or directly reduced the expression of genes responding to drought stress, and this could also be the reason that BMV-YC12-1 and BMV-YC12-5 are sensitive to drought.

## 4 Discussion

NF-Ys are TFs that regulate plant development and are involved in the flowering time, stress resistance, and photosynthesis. The NF-Y family has been systematically analyzed in other species at the whole genome level, but the evolutionary relationship between family members has not yet been systematically reported in maize. Most current studies of NF-Y TFs have been conducted in the model plant *Arabidopsis thaliana*, while their functions in other plants, especially in crop plants, have yet to be determined. In this study, we analyzed the evolutionary relationship of NF-Y family members at the whole genome level in maize, selected the core key drought resistance genes in the family through bioinformatics and biological experiments, and analyzed the mechanism of action of *ZmNY-FC12* in response to drought and rehydration treatments. These research results provide a reference for exploring the biological functions of maize NF-Y members and are also excellent gene resources for use in drought resistant molecular breeding.

### 4.1 Evolutionary analysis and classification of ZmNF-Y Genes

The NF-Y complex is composed of three subunits (NF-YA, NF-YB, and NF-YC), and these control the expression of target genes by combining with the CCAAT sequence in the promoter. With the rapid development of high-throughput sequencing and omics technologies, the NF-Y gene has been found in many plants, such as potato (Li et al., 2021), peach (Li et al., 2019), and *Triticum aestivum* (Stephenson et al., 2007). We identified a total of 52 NF protein sequences (16 ZmNF-YAs, 19 ZmNF-YBs, and 17 ZmNF-YCs) that represented the primary transcript. To further verify the authenticity of the ZmNF-Y protein, the NF-Y protein in Arabidopsis and rice was employed to compile an evolutionary tree together with ZmNF-Y. The results showed that the NF-Y protein of the three species was cross-distributed, and they formed a common mixed cluster. For example, *ZmNF-YB2* and *AtNF-YB2* were in the same branch while *ZmNF-FC8* and *OsNF-YC7* were in another branch, which further confirmed the correctness of NF-Y protein identification. The motif of ZmNF Y was significantly different in groups A, B, and C in this study. For example, motif 3 was unique to ZmNF-YA, while motif 5 was mainly present in ZmNF-YC. This phenomenon has also been found in potato (Li et al., 2021). The gene structure showed that ZmNF-Y members in the same branch of the evolutionary tree had a similar exon intron structure, while some ZmNF-Y members had no intron structure, which is consistent with findings in *Brassica napus* L. (Liang et al., 2014) and chickpea (Chu et al., 2018). The NF-YB subfamily *ZmNF-YB19* contained 26 exons, but its encoded amino acid sequence contained no motifs, but *ZmNF-YB19* has the largest number of exons and the largest protein molecular weight. High frequency of new mutations and heterotopic breakpoints are easy to occur when the number of gene bases is large, what’s more, the encoded high-quality protein has unstable characteristics. The largest number of exons and disordered amino acid sequences indicate that ZmNF-YB19 may have significant functional differences from other ZmNF-Y’s, and it is specified that the gene has evolved. The results of this study raise an interesting question: is the number of exons related to the protein expression time? The biological function of *ZmNF-YB19* in maize thus needs to be further explored.

Conducting a genome collinearity analysis is effective for studying the degree of evolution among species and whether there is a replication relationship between genomes. Gene replication is one of the main evolutionary forces in the genetic system and genome process (Moore and Purugganan, 2003). This study showed that there are more NF-Y gene pairs between maize, sorghum and rice, which are monocotyledons, but fewer gene pairs between monocotyledonous maize and the dicotyledonous Arabidopsis. These results indicate that the NF-Y protein is highly conserved in the evolutionary process. Similar NF-Y genes among different species may have similar biological functions or regulatory pathways. The high similarity and timely differences between similar gene sequences of different species provide infinite possibilities for the evolution and differentiation of species.

### 4.2 ZmNF-Y Genes play vital roles in maize growth, development, and response to abiotic stress

NF-Y_S_ participates in the regulation of plant growth, development, and stress resistance (Chu et al., 2018). The expression analysis of all ZmNF-Y members in 18 different maize organs and tissues revealed that *ZmNF-Y* genes in the same evolutionary branch had similar expression profiles; this indicates that they may participate in similar developmental processes. In addition, *ZmNF-YA5*, *ZmNF-YB11*, *ZmNF-YC7*, and *ZmNF-YC9* were expressed at high levels in the embryo, endosperm, and whole seed; and *ZmNF-YB2*, *ZmNF-YB11*, *ZmNF-YC7*, and *ZmNF-YC9* were expressed at high levels in the roots, stems, leaves, internode, silks, and tassels. However, expressions of *ZmNF-YA12*, *ZmNF-YB7*, *ZmNF-YB9*, *ZmNF-YB19*, and *ZmNF-YC3* were only detected in individual tissues (**Figure 4A**). These results show that the functions of *ZmNF-*Y gene have also undergone differentiation during the long evolutionary process. Previously studied homologous genes of other species, such as *StNF-YA8*, are reported to be induced by drought stress (Yang et al., 2016).The evolutionary tree and collinearity analysis of this study found that the evolution of NF-Y family members was relatively conservative (**Figures 1**, **3**). It has been reported that NF-Y transcription factors play crucial roles in plant development of other species and a variety of abiotic stress, including salt, drought, heat, and freezing (Petroni et al., 2012; Zhao et al., 2016). *PtNF-YA9* has been demonstrated to regulate seed germination, abiotic stress responses, and plant growth and development (Lian et al., 2018). The transcriptome data provided in this study show that some *ZmNF-Y* genes are associated with responses to drought and rewatering (**Figure 4B**). For example, *ZmNF-YB3*/*YB7*/*YB11*/*YB17*/*YC3*/*YC9*/*YC12* were found to be up-regulated in leaves after stress, but significantly down-regulated after rewatering. *ZmNF-YA8* and *ZmNF-YC6* were significantly down-regulated in leaves after stress, and significantly up-regulated after rewatering. *ZmNF-YA13* was significantly down-regulated in roots and leaves after stress, and up-regulated after rewatering; and *ZmNF-YA14* was significantly down-regulated in roots after stress but up-regulated after rewatering. These genes thus play important roles in the drought and rewatering treatments.

A WGCNA is a method commonly used to identify coexpression regulatory networks, and it has been shown to be highly effective in many studies (Zhao et al., 2021). The WGCNA conducted here showed that *ZmNF-YA8* and *ZmNF-YC6* responded to ABA under drought and rewatering treatments, *ZmNF-YB11* responded to the proline content, and *ZmNF-YC12* and *ZmNF-YC3* responded to SOD (**Figure 5A**). These results indicate that these genes play important roles in drought and rewatering treatments; thus, this study lays the foundation for exploring their mechanisms. A gene co-expression network analysis can reveal possible interactions among gene products by detecting similarities in gene expressions, which can clarify intergenic interactions and identify core genes. In this study, 11 core genes (*ZmNF-YC3*/*YC12*/*YC6*/*YA2*/*YB17*/*YB11*/*YC4*/*YC7*/*YC8*/*YC14*/*YB3*) were identified by this method (**Figure 5B**). The six key genes screened by WGCNA and gene co-expression network analysis were further analyzed under high temperature, drought, high salt, and ABA treatments. After treatment, the expressions of *ZmNF-YB3*, *ZmNF-YB11*, *ZmNF-YC9*, and *ZmNF-YC12* first increased and then decreased, and the expression of *ZmNF-YC12* showed the largest difference (**Figures 6A-D**). *AtNF-YA5*, which has a high sequence similar to *ZmNF-YA2* protein, has been reported to respond to drought and ABA treatments, and *AtNF-YA5* improves drought resistance by regulating the expression of stress response genes (Li et al., 2008). In *Arabidopsis thaliana*, the overexpression of *TaNF-YA10-1* decreased the sensitivity to ABA, but it significantly increased the sensitivity to salt stress, and the expression levels of stress related genes *AtABI5*, *AtRD29B*, *AtRAB18*, *AtCBF3* and *AtCBF1* decreased (Ma et al., 2015). However, the sensitivity of *AtNF-YA1* overexpression plants to ABA and high salt stress increased (Li et al., 2013). These results provide a molecular basis for drought-resistant breeding.

### 4.3 *ZmNF-YC12* plays an important role in drought resistance and the recovery ability of maize

In this study, *ZmNF-YC12* was found to belong to nuclear protein and was identified as a transcriptional activator (**Figure 7**). The *ZmNF-YC12* screened by WGCNA and the expression of *ZmNF-YC12* showed the largest difference under high temperature, drought, high salt, and ABA treatments. These results suggest that ZmNF-YC12 may respond to drought and rewatering through the ABA pathway. The function of *ZmNF-YC12* was further analyzed by silencing it and analyzing the subsequent physiological and biochemical indicators and the expression of response genes in mutants (BMV-YC12-1 and BMV-YC12-5) and wild-type (BMV) plants. Phenotypically, compared with BMV, the leaves of mutant withered, and the green color faded drastically after drought stress and after rewatering (**Figure 8A**). It has been reported that antisense silencing of *OsHAP3A* (*OsNF-YB2*) leads to a reduction in the chlorophyll content and the degradation of chloroplasts (Miyoshi et al., 2003). In addition, the overexpression of *TaNF-YB3* significantly increases the photosynthetic rate, chlorophyll content, and the early growth rate of plants (Stephenson et al., 2011), and the overexpression of *PdNF-YB7* enhances the photosynthetic rate of *Arabidopsis thaliana* and reduces water loss, which provides strong drought stress tolerance (Han et al., 2013). These previously obtained results are consistent with those of our study. After silencing *ZmNF-YC12*, the chlorophyll content and photosynthetic rate of maize decreased significantly (**Figures 8B, C**). MDA is one of the most important products of membrane lipid peroxidation, and its production can also aggravate membrane damage, and the RWC is a critical index that is used to indicate plant tolerance to drought stress (Griffiths et al., 2014). One study of potato showed that the MDA content in the strain overexpressing *StNF-YC9* was lower than that in WT, while the RWC was higher than that in WT, and the drought resistance of potato was significantly improved (Li et al., 2021). In addition, the overexpression of soybean *GmNF-YA3* in Arabidopsis reduced the leaf water loss and improved the plant’s drought resistance (Ni et al., 2013). In this study, the MDA content and the water loss rate in the silenced *ZmNF-YC12* maize strains were higher than that of BMV, and the RWC was significantly lower than that of BMV, which is consistent with these results (**Figures 8D–F**).

Drought stress increases the reactive oxygen species contents of plants and damages plant cells. A series of antioxidant enzymes (such as SOD, POD, CAT and APX) subsequently play scavenging roles, thus maintaining cell function and metabolic activities. Soluble proteins are important osmoregulation substances and nutrients, and their increase and accumulation can improve the water holding capacity of cells and protect the living materials and biofilms of cells. They are thus often used as an indicator for screening resistance. The accumulation of proline is mainly realized by strengthening synthesis and weakening degradation. P5CS is an important enzyme in the proline biosynthesis pathway (Verslues and Sharma, 2010). However, the proline content is not only related to the key enzymes of the synthesis pathway, but it also has a very important relationship with the enzyme ProDH, which controls the degradation. Reducing the activity of ProDH is known to be of great significance for regulating osmotic balance, preventing osmotic stress from causing damage to plants, scavenging free radicals, and protecting cell structure (László and Arnould, 2010). In this study, the overexpression of *ZmNF-YBl6* maintained the higher photosynthetic efficiency and stronger antioxidant capacity of the plant under stress conditions, stabilized the protein processing capacity, and improved the stress resistance of the plant. Overexpression of *ZmNF-YA1* significantly improved the drought resistance of maize, and the cell membranes of leaves were slightly damaged. In contrast, the leaves of the overexpressed strains accumulated greater amounts of osmoregulation substances, maintained a higher relative water content, and the antioxidant protection enzyme activity was significantly increased, thus improving the clearance of free radicals (such as reactive oxygen species) and reducing cell damage (Yang et al., 2022). The results of this study are consistent with previously reported results. There were no significant differences in SOD, POD, CAT and APX enzyme activities among strains before stress treatment, but after five days of stress, the enzyme activities of BMV-YC12-1 and BMV-YC12-5 were lower than those of WT, and these remained the same after rewatering. An expression analysis of four antioxidant enzyme genes (*ZmSOD*, *ZmpOD*, *ZmAPX*, and *ZmCAT*) showed that their change trends were consistent with their corresponding antioxidant enzyme activities (**Figures 9A–H**). The changes in the soluble protein and proline contents and antioxidant enzyme activity of all strains were the same at before, drought and rewatering treatments (**Figures 10A, B**). We further analyzed the activities of key enzymes for proline synthesis (P5CS) and degradation (ProDH). After stress, the P5CS and ProDH activities increased and decreased, respectively, in all strains. After rewatering, a reverse trend was seen (**Figures 10C, D**), which was the same as the change trend of proline content. A further analysis of the expressions of *ZmP5CS* and *ZmProDH* (**Figures 10E, F**) showed that the change trends were consistent with the corresponding enzyme activity. The abilities of the BMV-YC12-1 and BMV-YC12-5 mutant plants to absorb and maintain water under drought stress were lower than that of the wild-type plant. In addition, the cell osmoregulation substance contents were lower, and the activities of SOD, POD, CAT and APX were significantly reduced. ZmNF-YC12 is a transcription activator. We speculate that under drought stress, ZmNF-YC12 can directly or indirectly regulate the expression of downstream target genes, such as proline synthesis and degradation enzyme gene and antioxidant enzyme gene. So as to maintain the balance of cell osmotic potential, activate the antioxidant enzyme system, protect the cell membrane and improve the drought resistance of maize. After rewatering, the growth and development of plants can also be maintained through the compensation mechanism, which also indicates the recovery ability of the gene. The above results indicate that silencing *ZmNF-YC12* may reduce the activity of antioxidant enzymes and the proline and soluble protein contents of cells, damage the cell membrane, and cause maize to be sensitive to drought. These complex and diverse changes suggest that *ZmNF-YC12* has a complex role in the maize drought resistance mechanism.

The microarray results showed that *AtNF-YB1* did not regulate the drought response element binding protein at the transcription level, nor did it participate in the regulation of ABA-dependent drought response pathway, so it was inferred that *At NF-YB1* might regulate drought stress tolerance through a new drought stress response pathway (Nelson et al., 2007). However, it is known that *PdNF-YB7* enhances the drought resistance of poplar through the ABA-dependent pathway, the transcriptional expression of *CdtNF-YC* induced by salt stress requires the participation of H_2_O_2_, NO and ABA, and the overexpression of Bermuda grass *CdtNF-YC1* can enhance the drought and salt resistance of rice (Chen et al., 2015). *SnRK2.3* (cross non-fermenting-1-related protein kinase 2.3) sucrose non-enzymolytic protein kinase (SnRK) is a kind of Ser/Thr protein kinase that exists widely in plants, and it is also a key positive regulator of the ABA signal transduction pathway. This kinase can be activated in many ways, such as by ABA, osmotic stress, or phosphorylation stress-related transcription factors, which protect plants from dehydration or hypersalination. A promoter analysis of *RD29B* (dehydration-responsive protein 29B) showed that two ABA response elements (ABRE), *AREB1* and *AREB2*, both play transcriptional activator roles in the ABA-induced expression of *rd29B* (Uno et al., 2000). *DREB* (dehydration-responsive element-binding protein) transcription factor is a binding protein of a drought response element, which can be combined with a CRT/DRE (C-repeat/dehydration-responsive element) cis-acting element in the downstream gene promoter to regulate the expression of a series of anti-stress functional genes that depend on this cis-acting element, thus causing an increase in proline, soluble sugar, chlorophyll, and other contents. This enhances the plant’s response to drought, low temperature, and high salt (Li et al., 2011). It has been reported that the *PaDREB* gene in *Populus albaxp* is highly expressed in drought, high salt, low temperature, and ABA conditions (Zhai et al., 2012). In maize, two DREB genes (*ZmDREB1A* and *ZmDREB2A*) belonging to the DREB1 and DREB2 subgroups, respectively, were cloned and demonstrated to be upregulated in response to plant water stress (Qin et al., 2004). Distinct from Arabidopsis *DREB2A*, the *ZmDREB2A* expression in response to abiotic stress was regulated *via* an alternative splicing mechanism and the expressed protein was found to directly activate downstream gene expression (Qin et al., 2007). This study found that *ZmNF-YC12* was strongly up-regulated after ABA treatment. Before drought stress, there was no significant difference in the expressions of *ZmSnRK2.3*, *ZmRD29B*, and *ZmDREB1.3* genes between BMV and the BMV-YC12-1 and BMV-YC12-5 mutant plants. However, after drought stress, the expressions of the three genes in the BMV-YC12-1 and BMV-YC12-5 mutant plants decreased significantly but increased to some extent after rewatering (**Figure 11**). These results show that *ZmNF-YC12* positively regulates drought resistance and the recovery ability of maize through the ABA-dependent pathway. The next step, therefore, is to explore the mechanism behind its regulation of the drought and rewatering processes by analyzing its target genes and associated interaction network; such results would provide new ideas for drought resistance molecular breeding.

## 5 Conclusions

In the present study, a total of 52 ZmNF-Y genes were identified in the maize genome, and comprehensive analyses of the structural features, phylogeny, and expression profiles were conducted. Six core genes responding to drought stress were screened, and their expression changes under high temperature, drought, high salt, and ABA treatments were analyzed. The Gal4-LexA/UAS system and a transactivation analysis in yeast identified ZmNF-YC12 as a transcriptional activator. In addition, silencing ZmNF-YC12 reduced net photosynthesis, the chlorophyll content, the antioxidant system activation of superoxide dismutase, catalase, peroxidase, and ascorbate peroxidase, and the soluble protein and proline contents. It also increased the malondialdehyde content, RWC, and water loss rate, which weakened the drought resistance and recovery ability of maize. These results provide insights into understanding the evolution of ZmNF-Y family genes in maize and their potential role in genetic improvement.

## Data availability statement

The datasets presented in this study can be found in online repositories. The names of the repository/repositories and accession number(s) can be found below: BioProject. BioProject ID is PRJNA942991.

## Author contributions

LC and XL conceived and designed the experiments. LC, YP, FY, and CM performed the laboratory experiments. GW, AF, and LC performed the data analysis and interpretation. LC wrote the paper. All authors contributed to the article and approved the submitted version.
