# Supplementary material for: Genome-wide identification of NF-Y gene family in maize (Zea mays L.) and the positive role of ZmNF-YC12 in drought resistance and recovery ability
Source: Front Plant Sci. 2023 May 17;14:1159955. doi: 10.3389/fpls.2023.1159955 (PMC10229843; doi:10.3389/fpls.2023.1159955)
Supplement: Supplementary file 1 [file DataSheet_1.zip › Supplementary Figures.DOC]

**Supplementary Figure S1**.The expression level of ZmNF-YC12 in BMV (WT) and ZmNF-YC12 mutants (BMV-YC12-1~5).

**Supplementary Figure S2**.The Cis-acting element in 2000bp upstream of ATG of ZmNF-Y genes.Different colors represent different Cis-acting elements.

**Supplementary Figure S3**.WGCNA module trait correlation diagram. Each row represents a module, each column represents a trait, blue represents negative correlation, and red represents positive correlation.
